# 25-hydroxyvitamin D levels in patients with obstructive sleep apnea and continuous positive airway pressure treatment: a brief review

**DOI:** 10.5935/1984-0063.20190126

**Published:** 2020

**Authors:** Dimitra I. Siachpazidou, Vasileios Stavrou, Spyridon Zouridis, Eudoxia Gogou, Nicholas-Tiberio Economou, Chaido Pastaka, Chrissi Hatzoglou, Konstantinos I. Gourgoulianis

**Affiliations:** 1 Laboratory of Respiratory Sleep Disorders, Department of Respiratory Medicine, Faculty of Medicine University of Thessaly, Larissa, Greece.; 2 Laboratory of Cardio-Pulmonary Testing, Department of Respiratory Medicine, Faculty of Medicine, University of Thessaly, Larissa, Greece.; 3 Department of Respiratory Medicine, Faculty of Medicine, University of Thessaly, Larissa, Greece.; 4 Sleep Study Unit, Eginition Hospital, University of Athens Medical School, Athens, Greece.; 5 Department of Physiology, Faculty of Medicine, University of Thessaly, Larissa, Greece.

**Keywords:** Obstructive Sleep Apnea, Vitamin D, Continuous Positive Airway Pressure Treatment, Adults

## Abstract

The aim of the present study is to summarize the information available, to time, regarding the relationship between obstructive sleep apnea (OSA) and vitamin-D (vD) levels. Moreover, the association between vD deficiency and OSA severity will also be examined. At the end of the present study the possible advantageous effect of CPAP on vD-levels will be summarized. Extensive literature search was conducted in PubMed, Scopus, The Cochrane Library and Embase database. 13 articles were found concerning OSA and vD, of which 2 articles included treatment with a CPAP. Patients with OSA exhibit low levels of vD in the blood serum, and women present an even lower mean value than men. Lack of VD in blood serum seems to be related to the severity of the OSA syndrome, and to the short duration of sleep. OSA patients with concurrent metabolic syndrome exhibit lower serum vD-levels, as compared with those without metabolic syndrome. Long-term continuous positive airway pressure treatment (CPAP) treatment can increase vD-levels in male OSA patients while no change is observed in women. OSA patients demonstrate lower levels of vD in multiple studies. The severity of the OSA may be associated with vD-levels and deficiency, however more studies are needed to assess that relationship due to contradictions in current bibliography. CPAP can increase vD-levels in male patients. The relation between vD and OSA and/or CPAP is important but recent; therefore further research is needed about the exact relationship to be clarified. Also, the effect of gender hormones on vD regulation in OSA patients should be further investigated.

## INTRODUCTION

Obstructive sleep apnea (OSA) is a chronic condition characterized by repetitive collapse of the upper airway during the sleep, leading to intermittent hypoxia and recurrent arousals from sleep. It’s a prevalent disorder particularly among middle aged obese men, although its existence in women as well as in lean individuals is increasingly recognized^1^. In order to diagnose and/or quantify the severity of OSA, 2 indexes are commonly used: respiratory distress index (RDI) and more widely apnea hypopnea index (AHI)^2^. According to the current bibliography, the prevalence of OSA at ≥ 5 AHI is estimated at 9 to 38% while at ≥ 15 AHI it ranges from 6 to 17% in general population, with higher rates among men. Interestingly the prevalence of OSA was considerably higher in the elderly patient groups^3^. It is generally estimated that at least 20% of the general population has symptoms that are compatible with OSA^4^. However, even in countries where the syndrome is widely recognizable, it frequently goes undetected^2^^,^^4^. CPAP device is the current gold standard and a highly effective method, used to treat adult patients with OSA. Continuous positive airway pressure treatment (CPAP) restores the upper airway airflow, reduces AHI and normalizes the number of arousals. Although the benefits of CPAP treatment are conspicuous, the effectiveness of treatment mainly depends on the proper use of CPAP device and the patient’s tolerance to the treatment, which is frequently problematic^5^^-^8.

25-hydroxyvitamin D (Vitamin D, vD) is a fat soluble vitamin contained in various foods^9^. Up to 25% of vitamin concentration can be obtained by diet, sunlight exposure and dietary supplementation. However, other genetic factors are considered to contribute at a rate of 23 to 80%^10^. Vitamin D and its receptor are present in multiple human tissues, including the brain where vD is considered to play a protective role^9^^,^^11^. Vitamin D sufficiency is generally considered at blood levels over 20ng/mL^9^^,^^11^. Deficiency of vD is a crucial problem worldwide associated with multiple risk factors, including inadequate exposure to sunlight, aging population and obesity, among others^11^. A recent continuous body of evidence relates vitamin D, and mostly its deficiency, to OSA.

The consequences of low vD levels have been extensively investigated in the past and have been associated with multiple cardiovascular disorders, like hypertension and CHD. The nervous system may also be affected, with evidence that diseases like multiple sclerosis, amyotrophic lateral sclerosis, Parkinson’s but also Alzheimer’s may be linked with low vD levels. Immune system disorders have also been associated with abnormally low vD levels, with vD deficient population being more susceptible to pathogen infections. Moreover hematological malignancies with poor prognosis, have been linked to vD deficiency, and finally, low vD levels have also been noted in patients affected by gastrointestinal tract disorders like inflammatory bowel disease^12^.

The purpose of this review was to emphasize on the current knowledge about vD-levels in patients with OSA and CPAP treatment.

## METHODS

The choice of literature was done aiming at a comprehensive coverage of the topic during the period November 2017 to December 2018. The key words used were: obstructive sleep apnea and/or OSA, vitamin D, continuous positive airway pressure treatment and/or CPAP. The primary research that has been used in the present paper is scientific research, both qualitative and quantitative, which has been published in international scientific journals. The studies selected involved adult patients. Articles that included patients with co-morbidities, review articles and meta-analyses were excluded while the articles used were in English. After performing a literature search with the above keywords in PubMed, Scopus, The Cochrane Library and Embase database, 12 articles were found concerning OSA and vitamin D, of which only 2 articles included treatment with a CPAP.

## RESULTS

After performing a literature search with the above keywords, 13 articles were found concerning OSA and vitamin D (Table 1), of which only 2 articles included treatment with a CPAP (Table 2), all regarding adult population. All studies were recent.

**Table 1:** Presenting research results in patients with OSA and without CPAP treatment in vitamin D levels.

References	Subjects	Age (yrs)	BMI (kg/m^2^)	Method	Results
Bozkurt et al.^22^	A: noOSA (n=47)	A: 42.8±9.5	A: 29.2±4.9	PSG, the serum 25-OH-D concentration (ng/ml) was determined using a commercial RIA kit (Immuno-Biological Laboratories, Minneapolis, MN) with normal ranges of 11.1-42.9 ng/ml, and HbA1c was measured by turbidimetric assay.	↓ vD in OSA_severe_ (AHI≥15)
	B: OSA_mild_ (n=46)	B: 47.8±10.4	B: 29.0±4.1		
	C: OSA_moderate_ (n=47)	C: 49.8±10.6	C: 30.8±5.1		
	D: OSA_severe_ (n=50)	D: 49.7±10.4	D: 33.4±4.6		
Mete et al.^21^	A: OSA_group_ (n=150)	A: 47.2±8.7	A: 32.9±5.0	PSG, 25(OH)D was measured at Roche Elecsys E411/2010 autoanalyzer using 25(OH)D (Roche Diagnostics, Germany) kit with ECLIA (electrochemiluminescence immunoassay) method	↑ OSA is related to ↓ vD-levels
	B: Control_group_ (n=32)	B:46.9±8.1	B: 32.0±4.7		
Barceló et al.^14^	A: AHI ≤15 (n=105)	A: 54.0±12.8 B: 53.0±12.4 C: 50.9±12.4	A: 31.9±5.6	PSG, 25(OH)D levels were quantified in duplicate using a commercial enzyme immunosorbent assay kit (IDS, Boldon, UK) with a coefficient of variation of less than 10%	Inverse association of vD-levels with among patients with OSA
	B: AHI 16-30 (n=377)		B: 32.0±6.7		
	C: AHI>30 (n=344)		C: 30.4±5.5		
Erden et al.^20^	A: Control_group_ (n=43)	A: 45±14	A: 27.8±5.1	PSG, 25(OH)D was parameter determined using the ADVIA Centaur XP chemiluminescence immunoassay (Siemens Diagnostics, USA)	↑ OSA is related to ↓ vD-levels
	B: OSA_moderate_ (n=23)	B: 42±9	B: 29.4±4.0		
	C: OSA_severe_ (n=62)	C: 51±10	C: 32.7±6.1		
Kerley et al.^19^	A: noOSA (n=31)	A: 53±19	A: 32±8	PSG, 25(OH)D was measured using competitive chemiluminescence immunoassays (Dia-Sorin, Dietzenbach, Germany)	Widespread vD deficiency and insufficiency in Caucasian OSA patients
	B: OSA_mild_ (n=22)	B: 54±19	B: 31±8		
	C: OSA_moderate_ (n=19)	C: 57±17	C: 33±7		
	D: OSA_severe_ (n=35)	D: 55.5±17	D: 39±8		
Piovezan et al.^15^	A: noOSA (n=195)	52.0(9.1)	31.8	PSG, Using the ARCHITECT 25(OH)D chemiluminescent microparticle immunoassay	↓ sleep duration (<6h) and AHI (≥5) are independently associated with the risk of vD deficiency in an adult population
	B: OSA_mild_ (n=181)				
	C: OSA_moderate_ (n=132)				
	D: OSA_severe_ (n=149)				
Toujani et al.^17^	A: OSA_group_ n=92	A: 52.3±12.7	A: 36.2±6	PSG, The serum concentrations of vD were assayed with a radioimmunoassay kit	vD deficiency was widespread in OSA_severe_
	B: Control_group_ n=3	B: 45.7±14.7	B: 32±4.2		
Salepci et al.^18^	A: Patients with vD deficiency (n=132, AHI=31.5±28.6, d <20 ng/ml)	A: 48.3±11.7	A: 32.2±6.1	PSG, The serum concentrations of VD were assayed with a radioimmunoassay kit (DiaSorin, Stillwater, MN, USA)	vD-levels did not differ by OSA diagnosis status or severity
	B: Patients without vD deficiency (n= 49, AHI=32.1±3.6, d≥ 20 ng/ml)	B: 50.7±13.3	B: 30.4±4.8		
Archontogeorgis et al.^31^	A: OSAwith metabolic syndrome (n=55)	A=54.8±14.4	A=51.8 ±13.5	PSG, Serum concentration of 25(OH)D was determined using a commercial radioimmunoassay kit and the manufacturer's specifications (DiaSorin, Stillwater, MN)	Patients with OSA and metabolic syndrome exhibit lower serum vD-levels, as compared with those without metabolic syndrome
	B: OSA_without metabolic syndrome_ (n=52)	B=37.7 ±6.4	B=35.2 ±9.1		
Archontogeorgis et al.^13^	A: OSA_group_ (n=139)	A: 53.9±12.8	A: 35.9±6.9	PSG, The serum concentrations of VD were assayed with a radioimmunoassay kit (DiaSorin, Stillwater, MN, USA)	↓ vD-levels in OSA patients compared with non-apnoeic controls
	B: Control_group_ (n=30)	B: 44.9±12.8	B: 29.9±6.8		

Abbreviations: AHI: apnea hypopnea index; BMI: body mass index; HbA1c: hemoglobinA1c; OSA: obstructive sleep apnea; PSG: polysomnography study; vD: vitamin D (25-hydroxyvitamin D).

**Table 2:** Presenting research results in patients with OSA and CPAP treatment in vitamin D levels.

References	Subjects	Age (yrs)	BMI (kg/m^2^)	Method	Follow up	Results
Liguori et al.^24^	A: OSA_group_ (n=90)			PSG, Serum vD-levels were quantified by routine clinical laboratory methods (Vista, Centaur and Immulite Siemens Healthcare Diagnostic, Milan, Italy)	Use CPAP therapy for a 1-week period	We found that only in male OSA patients who appropriately use noCPAP therapy for a 1-week period (≥4 h/night, mean residual AHI<5 /h, OSA) vD increased to adequate serum levels. Surprisingly, female OSA patients did not show changes in serum vD concentrations after CPAP treatment.
	B: Control_group_ (n=32)					
Liguori et al.^25^	A: Whole OSA_group_ (n=39)	A:Baseline=54.9±13.8,	A: Baseline=33.5±8.4,	PSG, Serum vD-levels were quantified by routine clinical laboratory methods (Vista, Centaur and Immulite Siemens Healthcare Diagnostic, Milan, Italy)	1 year of useful CPAP treatment	In this study, we documented the significant positive effect of long-term useful CPAP therapy on vD status in middle-aged men affected by severe OSA. In particular, the significant improvement of vD status after one year of useful CPAP therapy was more evident in obese OSA patients. Consistently, OSA patients and particularly obese with respect to non-obese OSA patients more frequently recovered a sufficient vD status after CPAP treatment.
	Baseline and CPAP	CPAP=55.9±13.8	CPAP=33.3±7.9			
	B: Obese OSA (n=23) Baseline and CPAP	B: Baseline=57.9±11.6,	B: Baseline=38.6±7.4,			
	C:non-obese OSA (n=16) Baseline and CPAP	CPAP=58.9± 11.6	CPAP=38.3±7.0			
	D: Controls_group_ (n=10) Baseline and CPAP	C: Baseline=50.7±15.9,	C: Baseline=26.3±1.8,			
		CPAP=51.7±15.9	CPAP=26.2±1.5			
		D: Baseline=54.1±16.6,	D: Baseline=31±6.4,			
		CPAP=55.1±16.6	CPAP=31.7± 8.4			

**Abbreviations:** AHI: apnea hypopnea index; BMI: body mass index; CPAP: hemoglobinA1c; OSA: obstructive sleep apnea; PSG: polysomnography study; vD: vitamin D (25-hydroxyvitamin D).

In the study by Archontogeorgis et al.^13^, patients were divided into two groups (A: OSA_group with metabolic syndrome_, n=55 vs. B: OSA_group without metabolic syndrome_, n=52). Serum vD-levels were significantly decreased in group A, as compared with group B (18 ± 8.6 vs. 23.9 ± 14.1ng/mL, respectively, *p*=0.012). Barceló et al.^14^ study also found that OSA patients with concurrent metabolic syndrome exhibit lower serum vD-levels, as compared with those without metabolic syndrome. Piovezan et al.^15^ in their study, which concerned 657 individuals with OSA, noted that moderate and severe OSA as well as the short duration of sleep are associated with vitamin D deficiency in adults (*noOSA* = 195, OSAmild = 181, OSAmoderate = 132, OSAsevere = 149). However, after subgroup analysis, only patients older than 50 year old exhibited that trend.

Moreover, Archontogeorgis et al.^16^, screening 139 subjects with OSA and 30 healthy controls, vD deficiency was observed only in OSA patients. More specifically, vD-levels were 17.8 ± 7.8 and 23.9 ± 12.4ng/ml for OSA and controls respectively. There was also noted a statistically significant relationship between indices of severity of the syndrome and vD-levels. Toujani et al.^17^, in their study involving 42 patients with severe OSA and 30 healthy controls, noted that all patients with OSA_severe_ had inadequate vD in the blood serum. The mean value of vD-levels in the OSA group was 7.9ng/ml whereas in the control group the vD-levels reached 16.8ng/ml. Salepci et al.^18^, in 162 patients with OSA (OSA_mild_ n=52, OSA_moderate_ n=38 and OSA_severe_ n=72) observed that the majority of the OSA patients were deficient in vD. Vitamin D level was defined at 15.5 ± 11.6ng/mL and 134 patients (74%) met the criterion for vD deficiency (< 20ng/mL). However, the levels did not differ proportionally to the severity of the syndrome.

A survey conducted in Ireland in 106 middle aged male OSA patients (mild/moderate/severe, mean age 54.5 years, BMI = 34.3 kg/m^2^), it was found that 98% of OSA patients had insufficient vD (< 75nmol/L), including 72% with vD (< 50nmol/L). Vitamin D levels decreased with OSA severity in a statistically significant manner^19^. In another study of 128 patients newly diagnosed with OSA, divided them into three groups: control n=43, the OSA_moderate_ n=23 and the OSA_severe_ n=62, it was observed that vD-levels were deficient in all OSA groups and significantly lower when compared to the control group (Vitamin D: control = 29.5 ± 9.1ng/mL, OSA_moderate_ = 22.1 ± 7.2ng/mL and OSA_severe_ = 23.5 ± 7.7ng/mL)^20^. Moreover, in a study of 635 male and 191 female patients with OSA it was found that in 55.3% of men and in 63.2% of women, vD-levels were less than 30ng/ml. The authors also observed that patients with low vitamin levels tended to be older and have higher BMI values. Interestingly the same study did not observed a significant difference of vitamin D levels between groups of different OSA severity^14^.

The aim of another study by Mete et al.^21^ was also to investigate the relationship between Vitamin D levels and OSA severity. 150 patients with OSA (50 mild, 50 moderate and 50 severe disease) and 32 healthy controls were included in this study. This study also resulted in no statistically significant difference of vD-levels between patients with OSA (OSA: 17.91 ± 9.25 vs. controls: 19.17 ± 7.21ng/dl). However, in the subgroup analysis of OSA patients, it was noted that patients with OSA_severe_ (vD-levels: 14.66 ± 8.19ng/dl) had significantly lower levels compared to the mild (vD-levels: 20.65 ± 9.65ng/dl) and moderate group (vD-levels: 18.40 ± 9.02ng/dl)^21^. Finally, in a study of 190 OSA patients, lower vitamin D levels were found in respect to the control group (17.4 ± 6.9ng/dl vs. 19.9 ± 7.8ng/dl), while the reduction was found proportional to the severity of the syndrome. The same study concluded that females with severe OSA had significantly lower vD-levels (11.55ng/ml), while control males had the highest mean value (21.7ng/ml, *p*<0.001)^22^. On the other hand, a study conducted on elderly male patients (2.827 patients aged 76.4 ± 5.5 years) regarding the association between OSA and vitamin D deficiency, shows no significance (*p*=0.10). However, low levels of vitamin D were significantly associated with increased BMI and a large neck circumference. The authors suggested that low levels of vitamin D observed in OSA patients may be simply the result of greater BMI and neck circumference as a confounder^23^.

In the study by Liguori et al.^24^, comprising of 90 patients with OSA and 32 controls, after 7 days of proper CPAP treatment, patients with OSA show elevated levels of vD (mean OSA_severe_ vD = 19.34ng/ml vs. mean control group vD = 32.83ng/mL, *p*=0.0001). This finding applies only to male patients, since women with OSA did not exhibit any similar responses to CPAP treatment. The authors suggested that short-term CPAP treatment could promote vD-levels; however, that observation applies only in male patients. Finally, it was suggested that the influence of gender hormones in vitamin D regulation could be a possible explanation of vitamin D deficiency in women with OSA.

Another study conducted, aimed at assessing the effect of one-year use of CPAP on vD-levels in middle-aged OSA men. It was concluded that after one year of CPAP use, there was a significant increase in vD-levels. Hence, the authors suggested that long-term treatment with CPAP might be a viable treatment option to correct both sleep apnea and vD deficiency in middle-aged men with OSA. The phenomenon was more apparent in obese individuals and may represent a valid therapeutic strategy to ensure adequate levels of vD in that patient population, which often concurrently exhibits insufficient vD-levels^25^.

## DISCUSSION

The scientific community has recently begun to investigate the possible link between the 25-hydroxyvitamin D levels and OSA.

It is evident that OSA patients exhibit lower vD-levels, even when the control group is matched for important cofounders (such as BMI and age)^19^^,^^20^. Contradictory studies, though, are also present in the literature showing no confirmatory results^18^^,^^21^. Obesity has been frequently observed among those at risk for vD deficiency. In fact, OSA and vD deficiency share many relevant risk factors including age and obesity, which raises concerns for possible confounding factors to the associations being explored. Regarding obesity, several assumptions have been proposed, necessitating further investigation. For example obesity may result in lower vD-levels by reducing vD release or that the dietary habits resulting to obesity, may also have vD deficiency as a consequence^15^^,^^18^^,^^23^.

We found several data investigating the relationship between factors such as obesity, BMI, fat mass, body fat percent, metabolic syndrome and vD-levels and/or OSA presence, severity and AHI. The conclusions made are of great interest with metabolic syndrome being associated with lower vD-levels^13^^,^^14^, while higher BMI patients were found to have significantly lower levels of vD^14^^,^^19^^,^^20^^,^^26^^,^^22^. Multiple molecular mechanisms have also been proposed on how obesity is may be leading to lower vD levels. For example, the enzymes responsible for vD activation are not highly expressed in subcutaneous adipose tissue, probably leading to an overall increased catabolism of vD in excess body fat states. Other obesity associated factors such as adipokines and leptin levels have also been investigated but only contradictory data have been retrieved^27^^-^29.

Another aspect heavily investigated is the possible presence of a relationship between OSA severity, AHI scores and vD-levels. Some studies have repeatedly shown that there is probably no correlation between vD-levels and AHI score, even when AHI was measured in patients with severe OSA^17^^,^^18^^,^^21^. Two studies though, Kerley et al.^19^ and Goswami et al.^23^, demonstrated a negative, statistically significant correlation, between OSA severity level and vD. However, the latter one shows that the effect was diminished after adjustment for common OSA risk factors. It was assumed that the effect was probably due to the BMI effect^19^^,^^23^. In the contrary, the Kerley et al.^19^ study did not find BMI sufficient to explain the AHI-vitamin D correlation and suggested that OSA may induce lower vD-levels via a chronic inflammatory process or that low vD-levels predispose to exacerbations of OSA via inflammatory pathways. Several data may support that hypothesis. For example, vD supplementation has been associated with lower pro-inflammatory cytokine such as IL-17 levels while severe OSA patients exhibit a negative correlation between IL-17 and vitamin D^27^. Another interesting observation by Mete et al.^21^ study was that, although no association between AHI score and vD-levels was found, patients with severe OSA had significantly lower levels of vD-levels when compared to the controls. One very interesting hypothesis, though, based on the nocturnal intermittent hypoxia that OSA patients experience has been proposed in an effort to explain the link between vD levels and OSA in a molecular level. Having observed that Hypoxia Inducible Factor(HIF)-1a-subunit and Vascular Endothelial Growth Factor (VEGF) are conversely correlated with hypoxia indices in OSA patients^30^^,^^31^, and given the existing association between vD, HIF-1a-subunit and VEGF in human cancer cells but also the noted downregulation of HIF-1a-subunit, VEGF after CPAP treatment, those factors may also contribute to the lower vD levels of OSA patients^27^. Although plethora of data investigating those relationships has been gathered, the remarkable contradictions necessitate further analysis to reach to safe conclusions.

Another conclusion made by Piovezan et al.^15^ study was that besides the severity of the syndrome, short sleep duration is also directly associated with vD deficiency in adult patients with OSA. Of note is that similar conclusions are reached in the latest systematic review and meta-analysis by Neighbors, et al.^32^ regarding vD-levels differences between OSA and non-OSA groups and the negative correlation between vD-levels and OSA severity. However, the causal relationship between OSA and low vD-levels was unclear with the BMI consisting a possible confounding. Regarding the difference between the two sexes, despite the higher prevalence of OSA in males^1^, vD deficiency is more evident in women^14^^,^^22^.

Finally, concerning CPAP use, long-term treatment with CPAP has been proposed as a viable therapeutic option for correcting both sleep apnea and vD deficiency in middle-aged men with OSA, most evident in obese individuals. Liguori et al.^25^ study showed that short-term CPAP treatment could increase vD-levels in male OSA patients. The influence of gender hormones on vD regulation is a possible explanation of vitamin deficiency in women with OSA as it often affects postmenopausal women^24^.

### Limitations

Regarding the limitations of this literature review the search took place on the PubMed Scopus, The Cochrane Library and Embase databases. The articles that were used included the keywords: obstructive sleep apnea, vitamin D and CPAP. The search was age-targeted to adult patients. Articles that included comorbidities, literature review articles as well as articles of meta-analysis were excluded. Despite authors’ best efforts, it is possible that they failed to include some relevant studies. Each study analyzed has its own limitations, which may be added to those of this review.

## CONCLUSION

In conclusion, based on the already published studies, which are not very numerous, it seems that most studies show that patients with OSA exhibit lower levels of vD. The severity of the syndrome, the coexistence of metabolic syndrome and the short duration of sleep seem to be related to vD-levels in the serum of OSA patients, with lower mean values observed in female patients. However, further studies are needed to establish this observation. CPAP treatment may increase vD-levels in male OSA patients while women do not seem to exhibit similar response, and this may be the result of gender hormones. The influence of gender hormones on vD regulation is a possible explanation of vitamin deficiency in women with OSA as it often affects postmenopausal women. Further research is needed because, as mentioned before, until now only a limited number of studies have evaluated the relationship between OSA and vD-levels as well as the effect of treatments with a CPAP device.
